# Comparison of IPSA and HIPO inverse planning optimization algorithms for prostate HDR brachytherapy

**DOI:** 10.1120/jacmp.v15i6.5055

**Published:** 2014-11-08

**Authors:** Vanessa Panettieri, Ryan L. Smith, Natasha J. Mason, Jeremy L. Millar

**Affiliations:** ^1^ William Buckland Radiotherapy Centre, The Alfred Hospital Melbourne; ^2^ School of Applied Sciences, RMIT University Melbourne; ^3^ Alfred Health Radiation Oncology, The Alfred Hospital Melbourne; ^4^ Department of Epidemiology and Preventive Medicine School of Public Health, Monash University Melbourne Victoria Australia

**Keywords:** HDR brachytherapy, treatment planning, optimization algorithm, prostate cancer

## Abstract

Publications have reported the benefits of using high‐dose‐rate brachytherapy (HDRB) for the treatment of prostate cancer, since it provides similar biochemical control as other treatments while showing lowest long‐term complications to the organs at risk (OAR). With the inclusion of anatomy‐based inverse planning optimizers, HDRB has the advantage of potentially allowing dose escalation. Among the algorithms used, the Inverse Planning Simulated Annealing (IPSA) optimizer is widely employed since it provides adequate dose coverage, minimizing dose to the OAR, but it is known to generate large dwell times in particular positions of the catheter. As an alternative, the Hybrid Inverse treatment Planning Optimization (HIPO) algorithm was recently implemented in Oncentra Brachytherapy V. 4.3. The aim of this work was to compare, with the aid of radiobiological models, plans obtained with IPSA and HIPO to assess their use in our clinical practice. Thirty patients were calculated with IPSA and HIPO to achieve our department's clinical constraints. To evaluate their performance, dosimetric data were collected: Prostate PTV $D_{90}(\%),V_{100}(\%),V_{150}(\%)$, and $V_{200}(\%)$, Urethra $D_{10}(\%)$, Rectum $D_{2\text{cc}}(\%)$, and conformity indices. Additionally tumor control probability (TCP) and normal tissue complication probability (NTCP) were calculated with the BioSuite software. The HIPO optimization was performed firstly with Prostate PTV (${\text{HIPO}}_{\text{PTV}}$) and then with Urethra as priority 1 (${\text{HIPO}}_{\text{urethra}}$). Initial optimization constraints were then modified to see the effects on dosimetric parameters, TCPs, and NTCPs. HIPO optimizations could reduce TCPs up to 10%–20% for all PTVs lower than 74 cm^3^. For the urethra, IPSA and ${\text{HIPO}}_{\text{urethra}}$ provided similar NTCPs for the majority of volume sizes, whereas ${\text{HIPO}}_{\text{PTV}}$ resulted in large NTCP values. These findings were in agreement with dosimetric values. By increasing the PTV maximum dose constraints for ${\text{HIPO}}_{\text{urethra}}$ plans, TCPs were found to be in agreement with IPSA without affecting the urethral NTCPs.

PACS numbers: 87.55.‐x, 87.55.de, 87.55.dh, 87.53.Jw

## I. INTRODUCTION

Several authors have reported the benefits of using interstitial brachytherapy as an alternative to radical prostatectomy and external beam radiotherapy for the treatment of low and intermediate stage prostate cancer.^1^, ^2^, ^3^, ^4^ Results of multicenter studies^5^, ^6^, ^7^, ^8^ have shown that brachytherapy delivered as monotherapy or concurrently with external beam radiotherapy yields biochemical control rates similar to other techniques, while showing the lowest rates of long‐term complications to the organs at risk (OAR).^(9)^ High‐dose‐rate (HDR) brachytherapy, performed with remote afterloaders, has also the additional advantage of potentially allowing dose escalation^(10)^ without increasing considerably OAR toxicities or treatment times.

HDR is widely used, due to the recent ability to integrate 3D images into the treatment planning process. These images, which can be obtained either by computed tomography (CT) scans or ultrasound, provide the possibility to perform an accurate treatment plan based on the anatomy of the patient and the position of the implant at the time of treatment.

Additionally, the quality of HDR brachytherapy planning has advanced with the introduction of inverse planning optimizers similar to those used in external beam planning.^11^, ^12^, ^13^ These algorithms, which are now implemented in commercial treatment planning systems (TPS), generate reproducible treatment plans in a faster way by using clinical constraints set by the users.

Among the optimizers currently available, there has been great interest in the development and use of the Inverse Planning Simulated Annealing optimization algorithm (IPSA), in particular for the treatment of prostate cancer. IPSA is an anatomy‐based algorithm which optimizes the source dwell times using a simulated annealing algorithm, based on the work by Kirpatrick et al. ^(14)^ and developed for brachytherapy applications by Lessard and Pouliot.^(11)^ The model is governed entirely by the anatomy of the patient contoured from a CT scan and by a series of surface or volumetric prescribed dose constraints set by the user at the time of planning. IPSA gives an acceptable conformal plan in a matter of seconds by providing the distribution of the dwell times within the catheters. However, it was not initially designed to include a smoothness function to take into account the distribution of a single dwell time with respect to the adjacent ones.

**Figure 1:**
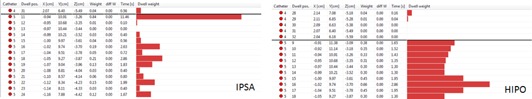
Example of dwell times distribution as calculated by IPSA (left) and HIPO (right) for the same patient and catheter.

The result of a standard unrestricted IPSA plan is that, in the majority of cases, the dwell times have an inhomogeneous distribution similar to the one shown in Fig. 1 (left) in which there are a number of dominating dwell times in particular positions within the catheter, usually at both ends, leaving the others with very small times or empty. This behavior could potentially lead to localized hot spots and, more importantly, to underdosage of the planning target volume (PTV) and overdosage of the OAR in cases in which there is a displacement of the catheters.^(15)^ Recently the Dwell Time Deviation Constraint (DTDC) parameter has been added to the IPSA optimizer implemented in the Oncentra Brachytherapy (OCB) treatment planning system (TPS) V. 4.3 (Nucletron B.V., Veenendaal, The Netherlands). This option can restrict the dwell time deviation in each catheter so as to control potential hot spots around individual dwell positions; however, its use is new and its effect is still under investigation.

As an alternative to IPSA brachytherapy, TPS users have started looking into different optimization approaches, among them the Hybrid Inverse treatment Planning Optimization algorithm (HIPO),^(13)^ which was also recently implemented in OCB V. 4.3, to be used for a variety of treatment sites including the prostate.

In the present work, a series of patients treated for low‐ and intermediate‐risk prostate cancer was retrospectively replanned with both the IPSA and HIPO algorithms implemented in OCB V. 4.3 for the same initial constraints. The resulting plans were then analyzed in order to evaluate the differences between them and the benefit of their use in clinical routine. Previously, HIPO was evaluated for the particular case of gynecological cancer^(16)^ and in comparison to geometrical and graphical optimization for HDR prostate brachytherapy.^17^, ^18^

## II. MATERIALS AND METHODS

### A. Clinical plans

Thirty patients treated between 2007 and 2008 were chosen from our institution's clinical database. These patients were all treated consecutively with CT‐based plans originally performed with the Plato V. 14.3.2 TPS (Nucletron) using geometrical optimization. The prostate planning target volume (PTV), rectum, and urethra were all contoured at the time of treatment by the same oncologist. The prostate PTVs covered a wide range, between 26 and 121 cm^3^.

According to the protocol followed at the time of treatment all patients were planned to receive 19 Gy in 2 fractions. All plans were exported from Plato and imported into OCB V. 4.3 TPS. This version of Oncentra allows the user to perform both manual and optimized planning on the reconstructed clinically placed catheters.

### B. IPSA optimization

Following our current clinical practice, the plans were first optimized with the IPSA algorithm using the initial parameters shown in Table 1.

**Table 1:** Initial IPSA optimization parameters used for the patients included in the study.

					*Surface*			*Volume*		
*ROI*	*Usage*	*Margin (cm)*	*Actv*.	*Weight*	*Min. (Gy)*	*Max. (Gy)*	*Weight*	*Min. (Gy)*	*Max. (Gy)*	*Weight*
Prostate	Target	0.32	0.50	100	9.5	14.25	100	9.5	14.25	30
Rectum	Organ	0.00	0.00			6.65	20			
Urethra	Organ	0.00	0.00	80	9.5	10.93	70			

As mentioned, the initial implementation of IPSA does not include a function which aims at adjusting the smoothness of the dwell time distributions within the catheters. The result is that in most cases after performing an IPSA optimization it is still necessary to adjust the dwell time manually to avoid high‐dose gradients (Fig. 1 (left)).

Since the DTDC parameter is currently under investigation and not clinically used in our institution, in order to perform a clinically relevant comparison, in this analysis the dwell times after an IPSA optimization were not manually modified and the DTDC parameter was disabled in order to have unrestricted optimization.

### C. HIPO optimization

Using the clinically placed needles, all patient plans were then calculated using the HIPO algorithm implemented in OCB V. 4.3. HIPO is a CT‐based 3D anatomy‐based algorithm^(13)^ which uses a combination of deterministic and stochastic models in order to potentially perform — the inverse optimization of needle placement (by a heuristic algorithm) and the inverse optimization of dwell time for a given needle or applicator configuration (quasi‐Newton algorithm). In this work, only the second option was used and HIPO plans were obtained by assigning dosimetric constraints similar to those used for the IPSA plans, as shown in Table 2.

**Table 2:** Initial HIPO optimization parameters used for the patients included in the study.

*ROI*	*Usage*	*Min. weight*	*Min. Value (Gy)*	*Max. Value (Gy)*	*Max. Weight*	*Priority*
Prostate	Target	100	9.50	14.25	100	2
Rectum	Organ			6.65	20	
Urethra	Organ			10.93	70	1

HIPO requires only the use of volumetric constraints, but allows setting optimization priorities to the target and OAR. In this study, for each patient two plans optimized with different HIPO settings were carried out: the first was done by assigning priority 1 to the Prostate PTV (defined as ${\text{HIPO}}_{\text{PTV}}$ in the text) and the second by assigning priority 1 to the urethra (${\text{HIPO}}_{\text{urethra}}$) in order to observe the effect of this parameter on the overall plan.

HIPO also allows the users to lock a number of catheters in order to keep their dwell times fixed and perform the optimization of the remaining catheters. This option, which has been widely used in gynecological applications, aims at restricting modulation and eliminating hot spots. In addition, it also offers the option of a modulation restriction (MR) parameter, which allows the user to obtain control of the free modulation of the dwell times in order to have smoother source movement and dwell time distribution within the catheters. However, as shown in previous works,^(17)^ it does not seem to introduce major improvements for prostate HDR cases. In this work, both options were disabled to perform a direct comparison with the IPSA optimizer.

To assess the effect of changing the initial HIPO optimization constraints, ten patients were then recalculated by changing the prostate PTV maximum initial constraint (Max Value(Gy)) from 14.25 Gy to 18 Gy.

### D. Analysis

All patient plans performed with IPSA, ${\text{HIPO}}_{\text{PTV}}$, and ${\text{HIPO}}_{\text{urethra}}$ were evaluated by comparing dosimetric parameters, radiobiological parameters, and global conformity indexes.

The dosimetric parameters analyzed were the dose‐volume histograms (DVH)‐based values proposed by GEC/ESTRO‐EAU^(19)^ for the Prostate PTV: the dose that covered 90% of the volume $D_{90}(\%)$, the percentage of the prostate PTV that received at least 100% of the prescribed dose $V_{100}(\%)$, the volume that received 50% and 100% more than the prescribed dose $V_{150}(\%),V_{200}(\%)$, and for the OARs the dose that covered 10% of the urethra $D_{10}(\%)$ and the dose that covered 2 cm^3^ of the rectum $D_{2\text{cc}}(\%)$. According to clinical practice, acceptability of the plan was evaluated according to the values provided in Table 3. Statistical significance between different algorithms was proven with a two‐sided *t*‐test (α=0.05).

**Table 3:** Dosimetric parameter tolerances expressed as a percentage of the prescribed dose.

*Parameter*	*Acceptable*	*Not Acceptable*
$V_{100}(\%)$	90%–100%	0%–84%
$V_{150}(\%)$	10%–32%	≥36%
$V_{200}(\%)$	3%–8%	≥12%
$D_{90}(\%)$	89%–119%	≥119%
Urethra $D_{10}$ (%)	0%–110%	≥115%
Rectum $D_{2\text{cc}}(\%)$	0%–66%	≥70%

Dosimetric parameters are obtained by using DVH calculated by the TPS for each structure. The DVH is extremely dependent on the size of the histogram bin and its relative height, and this variability can directly influence the dosimetric parameter calculated. For this reason, comparisons were also made by considering radiobiological indexes for both PTV and OAR, namely tumor control probability (TCP) and normal tissue complication probability (NTCP). These parameters were calculated by employing BioSuite,^(20)^ a software tool specifically designed for radiobiological analysis. TCP values were obtained by using a Poisson model,^(21)^ while NTCP parameters were obtained by using a Lyman‐Kutcher‐Burman (LKB) model.^22^, ^23^ Since there is much discussion on the appropriate parameters to be used in order to model tumor control for prostate cases,^24^, ^25^, ^26^, ^27^, ^28^ different combinations of modeling values were used in this analysis. As previously performed by Uzan and Nahum,^(20)^ the α/β ratio was varied between 5 and 1.5 Gy. Despite the general belief that the α/β ratio should be low for these types of tumors, the value of 5 Gy was also considered, since several authors have highlighted the possible effect of hypoxia or dose heterogeneity in the assessment of α/β for prostate cancer.^25^, ^28^

The other parameters, such α and α‐ spread were assigned accordingly.^(20)^ Additionally the clonogen density^(25)^ was varied between 10^5^ and 10^7^. Tumor repopulation was not considered for these types of diseases as they repopulate very slowly. To determine the best set of parameters, an average TCP was considered according to the collected clinical data at our institution. This value was considered to be between 70%–80%, assuming an average of five years biochemical tumor control for each patient.

In order to model NTCP for the OAR, rectal bleeding was considered the endpoint for the rectum. According to the QUANTEC publication,^(29)^ the parameters were set to be α/β=3Gy,n=0.09 for volume effect, m=0.13, and ${\text{TD}}_{50}=76.9\;\text{Gy}$. These values were confirmed by Liu et al.^(29)^ and were considered suitable for this cohort of patients. For the urethra, NTCPs were estimated by looking at shrinkage, ulceration, and stricture. In contrast to the rectum, parameters to model urethral complications are not readily available and, again, there is not a general consensus on the most appropriate values to be used for prostate HDR brachytherapy. In this work they were set to α/β=5Gy,n=0.085 for volume effect, m=0.27, and ${\text{TD}}_{50}=60\;\text{Gy}$, according to the recent publication by Gloi and Buchanan.^(27)^ These parameters provided an average urethral NTCP of 25% in accordance to our institution's collected clinical data.

Finally, in order to look at the quality and homogeneity of the plans, the conformation number (CN) proposed by van't Riet et al.^(30)^ and the conformal index defined by Baltas et al.^(31)^ (COIN) were also compared.

## III. RESULTS

### A. Dosimetric parameters

Mean and standard deviation values of the dosimetric parameters obtained for Prostate PTV and OARs are presented in Table 4. The last two columns represent the statistical significance of the differences between doses calculated with IPSA and, respectively, ${\text{HIPO}}_{\text{PTV}}$ and ${\text{HIPO}}_{\text{urethra}}$. According to the *t*‐test and taking IPSA as the reference algorithm, differences between IPSA and ${\text{HIPO}}_{\text{PTV}}$ and ${\text{HIPO}}_{\text{urethra}}$ were all statistically significant, except $V_{150}(\%)$ for ${\text{HIPO}}_{\text{PTV}}$.

**Table 4:** Comparison of mean and standard deviations of all the dosimetric parameters analyzed in the study.

		*IPSA*		${\text{HIPO}}_{\text{PTV}}$		${\text{HIPO}}_{\text{urethra}}$		*p*	*p*
*Parameters*		*mean*	*SD*	*mean*	*SD*	*mean*	*SD*	$\text{IPSA}/{\text{HIPO}}_{\text{PTV}}$	$\text{IPSA}/{\text{HIPO}}_{\text{urethra}}$
PTV	$V_{100}(\%)$	97.8	2.4	90.6	4.7	88.2	5.8	≤0.001	≤0.001
PTV	$V_{150}(\%)$	23.7	6.8	21.8	3.4	18.9	2.7	0.094	≤0.001
PTV	$V_{200}(\%)$	8.7	1.9	7.9	0.9	7.3	0.8	0.018	≤0.001
PTV	$D_{90}(\%)$	109.1	5.0	101.4	4.5	99.0	4.3	≤0.001	≤0.001
Urethra	$D_{10}(\%)$	112.1	4.7	126.9	10.9	109.3	2.8	≤0.001	0.003
Rectum	$D_{2\text{cc}}(\%)$	62.2	8.8	52.7	9.2	52.5	9.0	≤0.001	≤0.001

**Figure 2:**
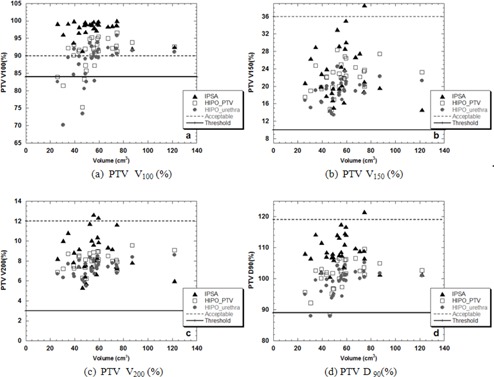
Values of PTV $V_{100}(\%)$ (a), $V_{150}(\%)$ (b), $V_{200}(\%)$ (c), and $D_{90}(\%)$ (d) calculated with IPSA (triangles), ${\text{HIPO}}_{\text{PTV}}$ (rectangles), and ${\text{HIPO}}_{\text{urethra}}$ (circles) as a function of the PTV.

Generally both HIPO optimizations yielded lower values of $V_{100}(\%)$ than IPSA independently of the size of the volume treated (Fig. 2(a)).

**Figure 3:**
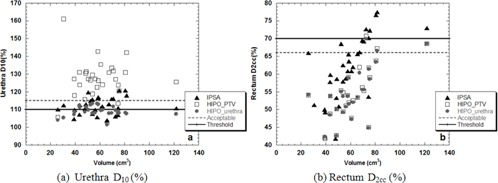
Values of $D_{10}(\%)$ (a) for the urethra and $D_{2\text{cc}}(\%)$ (b) for the rectum calculated with IPSA (triangles), ${\text{HIPO}}_{\text{PTV}}$ (rectangles), and ${\text{HIPO}}_{\text{urethra}}$ (circles) as a function of the volume of the PTV.

Considering each patient independently, in six patients, HIPO plans produced PTV $V_{100}(\%)$ below the clinical tolerances, summarized in Table 3 (as shown in Fig. 2). For these cases, it was not possible to find a correlation with the size of the PTV. Parameters related to inhomogeneity $V_{150}(\%)$ and $V_{200}(\%)$ were, instead, generally within acceptable limits (Figs. 2(b) and (c)), similar to $D_{90}(\%)$ which was within tolerance levels in all but two cases (Fig. 2(d)). Looking at the OAR for all patients analyzed, the urethra $D_{10}(\%)$ exceeded the acceptable tolerances for plans calculated with ${\text{HIPO}}_{\text{PTV}}$ in the majority of the cases. Considering the rectum, both HIPO calculations provided lower doses $\left(D_{2\text{cc}}(\%)\right)$ than IPSA (Figs. 3(a) and (b)).

### B. Radiobiological analysis

**Figure 4:**
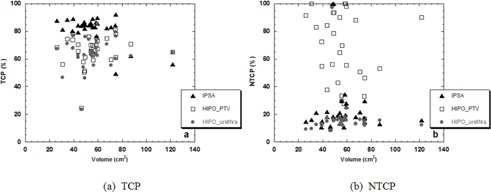
TCP (a) and NTCP (b) values calculated with IPSA (triangles), ${\text{HIPO}}_{\text{PTV}}$ (rectangles), and ${\text{HIPO}}_{\text{urethra}}$ (circles) DVHs as a function of the volume of the PTV.

Figure 4 illustrates the TCP and NTCP values obtained with the three types of optimization studied. For the OAR, only the urethra NTCP is shown since, as expected, the rectum NTCP was found to be negligible for all algorithms. For the TCP, the results shown are those obtained with α/β of 1.5 Gy and a clonogen density of 10^5^. For these parameters, the IPSA TCPs were between 70%–80%, which was the value expected from the clinical biochemical data.

The results show that the use of HIPO optimized with the same initial dosimetric constraints used in IPSA could potentially reduce the tumor control probability up to an average of 10%–20% for ${\text{HIPO}}_{\text{PTV}}$ and for ${\text{HIPO}}_{\text{urethra}}$ for all volumes lower than 74 cm^3^. Interestingly, this behavior changes for PTVs larger than 74 cm^3^, in both cases analyzed, as both HIPO algorithms provided TCPs 10% larger than IPSA.

For the urethra, the results show that IPSA and ${\text{HIPO}}_{\text{urethra}}$ provided similar NTCPs for the majority of cases and volume sizes, with ${\text{HIPO}}_{\text{urethra}}$ generally being lower than IPSA. Instead ${\text{HIPO}}_{\text{PTV}}$ resulted in large NTCP values, as expected from the dosimetric data (Fig. 4). Only in one case were IPSA and ${\text{HIPO}}_{\text{urethra}}$ larger than ${\text{HIPO}}_{\text{PTV}}$.

**Figure 5:**
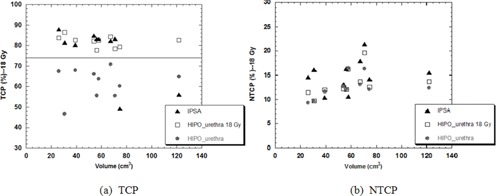
TCP (a) and NTCP (b) values calculated with IPSA (triangles), ${\text{HIPO}}_{\text{urethra}}$ 18 Gy (rectangles), and ${\text{HIPO}}_{\text{urethra}}$ (circles) DVHs as a function of the volume of the PTV.

Looking at a subset of patients with various size PTVs, if the initial prostate PTV maximum constraint was increased to 18 Gy, ${\text{HIPO}}_{\text{urethra}}$ provided TCP similar to IPSA without increasing urethral NTCP (Fig. 5).

### C. Conformity indices

**Figure 6:**
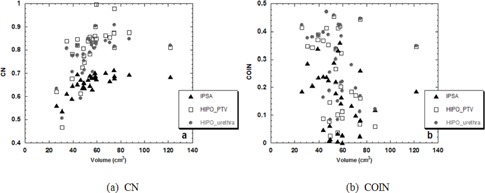
CN (a) and COIN (b) calculated with IPSA (triangles), ${\text{HIPO}}_{\text{PTV}}$ (rectangles), and ${\text{HIPO}}_{\text{urethra}}$ (circles) as a function of the volume of the PTV.

The CN and COIN values for each plan calculated using each of the three optimizations are illustrated in Fig. 6. The CN values show that the HIPO plans provided better conformation to the target volume than the IPSA plans, regardless of the target size. This behavior was generally confirmed by the COIN parameter, which also proved that HIPO plans typically tended to provide a larger degree of protection of the critical organs as well as target coverage.

## IV. DISCUSSION

### A. Planning target volume

The IPSA optimizer is widely used in HDR brachytherapy planning. However, its standard unrestricted implementation is known to provide plans usually characterized by large dwell times at the ends of each catheter (Fig. 1 (left)).^16^, ^32^ This behavior could lead to large delivery errors in the case of catheter movement, by significantly underdosing the target or potentially overdosing the OARs. For plans obtained with IPSA, in order to control such hot spots, it is common for the user to manually limit the large dwell times and then proceed to a final dose distribution using graphical optimization. All these steps increase the overall planning time and make treatment planning process less reproducible and robust.

For HDR prostate patients this analysis showed that the HIPO optimizer implemented in OCB V.4.3, used with 3D CT images and clinically placed needles, could provide a valid alternative to IPSA as it allowed production of an acceptable plan directly with inverse optimization, as previously seen for gynecological cases.^(16)^ Moreover it generally tended to provide more homogeneous dwell time distributions (Fig. 1 (right)).

The analysis of the dosimetric parameters recommended by GEC‐ESTRO^(19)^ showed that plans obtained with HIPO using the same initial parameters employed in IPSA provided lower $V_{100}(\%)$ and $D_{90}(\%)$ to the PTV, with an average difference within 7%–10%. Similarly $V_{150}(\%)$ and $V_{200}(\%)$ were lower, but the differences were of the order of 1%–4% (Table 4). Besides the dosimetric parameters being lower than IPSA, in six cases these values were below the clinical tolerances used in our department (Table 3 and Fig. 2).

These dosimetric results are directly reflected in the TCP parameters, but differences are within larger ranges (Fig. 4), since the TCP parameter is also very strongly correlated to the volumetric dose distribution in the target, represented by the differential DVH used for its calculation. In the majority of instances, plans calculated with HIPO showed lower minimum doses than those obtained with IPSA. This behavior could be due to the general tendency of HIPO to be more conformal to the target and more protective to the OARs, as shown by the CN and COIN values (Fig. 6). This trend could also be as a result of the different implementations of the two optimization algorithms and the use of the weights assigned to the various objectives in the final total objective function.

Due to the variety of radiobiological parameters associated with prostate TCP modeling in the literature,^24^, ^25^, ^26^, ^27^, ^28^ in this study various combinations were tested in order to match the average biochemical control recorded in ten years of HDR data collection at our institution. A lower α/β value of 1.5 Gy and clonogen density of 10^5^ appeared to reproduce, on average, the observed control of 70%–80%, confirming the hypothesis that a lower α/β ratio could be more appropriate to model its TCP.^(26)^ For prostate HDR brachytherapy, this result seems to be in accordance with the fact that large α/β ratios produce steeper dose response curves that are more sensitive to the large dose gradients characterizing these types of treatments.

Dosimetric values might suggest that simple rescaling of the initial parameters could provide HIPO plans dosimetrically equivalent to those obtained with IPSA. In order to confirm these findings, ten patients with different PTV sizes were recalculated with ${\text{HIPO}}_{\text{urethra}}$ by assigning as initial parameter a PTV maximum dose (Max Value (Gy)) of 18 Gy. All plans provided dosimetric parameters within the tolerances accepted (Table 3), and TCP values within 70%–80% expected by the clinical outcomes, while keeping NTCP values as low as the original IPSA plan (Fig. 5).

Interestingly, a detailed evaluation of individual patients also showed differences in dose distributions according to the size of the PTV volume (Figs. 2 and 4). For PTVs larger than 74 cm^3^, both HIPO algorithms provided better coverage and TCP than IPSA without any adjustment of the initial parameters. This result shows the potential benefit of using HIPO plans for treating patients with larger prostates, but in our cohort of patients only four cases had such large volumes, so more research is warranted to confirm this finding.

### B. Organs at risk

The HIPO optimizer available in OCB V. 4.3 allows assigning priorities to PTV and OARs, additionally to setting dosimetric constraints. If there is an intersection of volumes, the volume with the lower priority value is taken into account for generating dose points in the intersection. For example, if the PTV is set as priority 1 and the urethra is set as priority 2 and fully contained in the PTV, the class solution will not take into account the constraints set on the urethra, as this OAR will be considered part of the PTV. If instead the priorities are reversed, the urethra will be considered the organ with the highest priority to optimize. In our analysis, both options were considered in order to see the differences in the final dose distribution. As expected, for all patients, ${\text{HIPO}}_{\text{PTV}}$ plans provided lower dose coverage to the target than IPSA, but higher than those obtained with ${\text{HIPO}}_{\text{urethra}}$. However, the calculated urethral doses almost all exceeded the clinical tolerances, and were considered unacceptable for treatment. ${\text{HIPO}}_{\text{urethra}}$ instead in all cases was able to keep urethral doses equal to or lower than IPSA. From the NTCP analysis, the results again were confirmed; however, in two patient plans, the HIPO generated urethral NTCPs were significantly higher than with IPSA (Fig. 4). For these two cases, the differences could be attributed to the dose distributions represented in the DVHs, which showed large $V_{100}(\%)$ despite being in tolerance according to the $D_{10}(\%)$ value. For the subset of ten patients recalculated with a larger initial PTV maximum dose constraint, the urethral dose was still within tolerances (Table 3 and Fig. 5) and the NTCP was not significantly affected, proving that the ${\text{HIPO}}_{\text{urethra}}$ optimizer could be used with larger initial constraints to improve PTV coverage without affecting OAR sparing.

Rectal doses calculated with HIPO were in all cases lower than with IPSA, as shown in Table 4 and Fig. 3(b), showing that changing the algorithm would not increase the risk of toxicity for this organ.

## V. CONCLUSIONS

Prostate HDR brachytherapy benefits from the use of inverse planning performed by dedicated optimization algorithms. In this work, the widely used IPSA algorithm was compared with the HIPO algorithm, recently implemented in the OCB (V. 4.3). This analysis showed that HIPO used with priority 1 set to the urethra, could provide an alternative to IPSA and equally acceptable clinical plans if the initial maximum dose constraints are increased with respect to those used in IPSA, while providing a more conformal plan and, potentially, a more homogeneous distribution of the dwell times, possibly limiting the amount of hot spots in the dose distribution.

## ACKNOWLEDGMENTS

The authors acknowledge Peter Douglas and Kirsten Bell (Nucletron) for providing the new version of OCB (V. 4.3), and Karen Scott for providing the biochemical control data. VP gratefully acknowledges Alan Nahum and Julien Uzan for providing the software, BioSuite, and valuable feedback on the radiobiological analysis.
